# The role of local and regional authorities in prevention and control of NCDs: the case of Poland

**DOI:** 10.1186/s12914-020-00238-8

**Published:** 2020-07-22

**Authors:** Robert Tabaszewski

**Affiliations:** Department of Human Rights and Humanitarian Law, The John Paul II Catholic University of Lublin, Al. Raclawickie 14, 20-950 Lublin, EU Poland

**Keywords:** NCDs, Civilisation diseases, Determinants of health, Human right to health, Local and regional authorities, Risk factors, Health paradox, Poland, Health systems

## Abstract

**Background:**

Freedom from noncommunicable diseases is a component of the human right to health. The obligation to reduce the pandemic of civilisation diseases should result from the provisions of the constitution, national law and local regulations. This means that representatives of local and regional communities also take responsibility for limiting the pandemic of civilisation diseases.

**Main body:**

The goal of this debate is to examine the effectiveness of the decentralised polish model for dividing the competences of preventing and combating non-communicable diseases into three levels: local, regional and central. The representatives of regional and local communities are responsible for encouraging the inhabitants of their communities to change their lifestyle: eating habits, increase physical activity, remain outdoors more frequently, reduce stimulants, and apply prevention, namely undergo regular medical check-ups and provide the body with all the necessary nutrients. Limiting the proliferation of noncommunicable diseases also requires, in vertical terms, sound financial efforts on the part of local authorities. In the example of Poland, the claim about the effectiveness of the multi-level governance model which is presented in the literature will be verified.

**Conclusion:**

The excessive division of competences and the dispersion of resources between regional and local authorities may hamper the effective treatment and prevention of NCDs. A lack of cooperation between the central government, which carries out an independent policy on public health, and local authorities, which use only a portion of the public funds allocated to health, obstructs the debate on priorities at the societal level and does not encourage residents to take a stand on how to allocate extra funds.

## Background

Freedom from noncommunicable diseases (NCDs) is an undoubted component of the universal human right to health [1]. Currently, numerous international law instruments oblige national authorities, at both central and regional levels, to take appropriate measures to prevent and reduce the risk of civilisation diseases: cancer, cardiovascular diseases, allergies or diabetes [2]. The World Health Organization (WHO) understands this right to health not only as freedom from the above-mentioned diseases, but also the possibility for every person to use all available means that will allow achieving optimal health, free of addictions and negative determinants of health [3]. Europeans are also particularly vulnerable to factors that favour the occurrence of NCDs [4]. According to the WHO Regional Office for Europe, progressive environmental degradation, stress and sedentary lifestyles, poor nutrition and even poorer quality of food products cause regular deterioration of living conditions [5]. Paradoxically, with the development of society, natural, ecological, chemically clean food products can be expensive and are more and more difficult to get [6].

The primary goal of this debate is to illustrate the need for local and regional communities to take responsibility for limiting the potential for pandemics of civilisation diseases by preventing and controlling noncommunicable diseases (NCDs). Especially in countries of so-called systemic transformation, including Poland, there is no relationship between the lack of implementation of health strategies at a local level and the intensification of the NCD pandemic. This is also confirmed by data from the nationwide survey of the population’s health condition. Based on the data, it was established that 42% of men and 25% of women in Poland smoke cigarettes, 61.6% of men and 50.3% of women suffer from obesity, and 50% of adult Poles have low physical activity [7]. NCDs are estimated to account for 90% of all deaths [8]. Unfortunately, the role of local and regional authorities in the prevention and control of NCDs as a scientific and normative concept is largely absent from the literature. The role of local and regional authorities in the prevention and control of NCDs should be considered not only as a theoretical issue, but also as an empirical fact that needs public and academic exploration.

In addition to the WHO Constitution, the basic standard of human freedom from factors influencing individual and social health is determined by the International Bill of Rights: Art. 25, section 1 of UDHR [9], Art. 7 of ICCPR [10] and Art. 12 of ICESCR [11]. Human Rights Committee confirmed the freedom from factors increasing the incidence of NCDs in the André Brun v. France case [12]. The obligation of national authorities to prevent NCDs at all levels is also indirectly confirmed by all regional instruments. In the European system, freedom from endemic and pandemic diseases is defined explicite in Art. 35 of CFREU [13] and Art. 11 of ESC [14]. This last provision imposes an obligation on national authorities at all levels to undertake preventive actions against the effects of NCDs (the first and the third objectives), and then obliges them to provide a certain protection standard [15]. This obligation is addressed to public authorities, understood not only as a government but all entities, bodies and institutions with a state or local government [16].

A main motivating factor for this debate is the fact that the obligation to reduce pandemics of civilisation diseases should also result from the provisions of a country’s constitution and national laws. In this context, the main goal of this debate is to examine the effectiveness of the domestic model for dividing the competences of preventing and combating NCDs. Although, currently the right to health is a universal right contained in 88% of the constitutions of countries, only a few constitutions oblige national authorities to prevent NCDs [17, 18]. In the case of Poland, the addressees of the obligation expressed in Art. 68 of the Constitution of the Republic of Poland that guarantees every person the right to health care are all entities with a state or local government character. This means that representatives of local communities also take responsibility for limiting the pandemic of civilisation diseases [19]. In the case of Poland, these are local institutions: 2478 communes and 380 poviats, as well as regional institutions: 16 provinces [20]. The functioning of these three types of local government as a form of horizontal decentralization is in accord with the ideas of local democracy, local autonomy, local governance, decentralization and new localism [21, 22].

In this context the main aim of this debate is to examine the effectiveness of the domestic model for dividing the competences of preventing and combating NCDs. The Polish model of health protection is based on the concept of multi-level governance, which implies the simultaneous participation of all entities of public and social life, politicians and representatives of central, regional and local administrations in ensuring the protection of human health, as well as stipulating the involvement of the local council of doctors, nurses and patient and consumer organizations [13, 21]. The idea of multi-level governance in the healthcare system necessitates the simultaneous use of many concepts: decentralization, localism, sustainable development in healthcare, freedom from central interference, horizontal cooperation between regional and local government units and, above all, the principles of good governance [22, 23].

I undertook a critical review of the legal, political, and medical literature, analysed its content, and identified key lines of this issue. In this debate, I describe the need for cooperation between the central government and local and regional authorities, and “indicate the ability of local and regional authorities to make and enforce rules and to deliver services, regardless of whether that government is, or is not, democratic.” In this debate, I describe the need for cooperation between the central government and local and regional authorities and indicates the ‘ability to make and enforce rules, and to deliver services, regardless of whether that government is democratic or not’ [23–25]. This mechanism presupposes the need for cooperation at the central, local and regional levels, for the mutual trust of all entities, for the recognition of their competences and for the existence of a common goal: preventing and combating the effects of NCDs. Finally, I will conclude with practical recommendations on how the division of competences and the dispersion of resources between regional and local authorities may hamper the effective treatment and prevention of NCDs.

### Local and regional authorities’ responsibility for NCDs

The concept of the multilateral coordination model between local and regional authorities and central government has been affirmed by the Council of Europe [14, 16, 18]. The ESC imposes obligations on the state for securing the individual and the whole of society against the possibilities of health issues. According to the ECSR, the state’s tasks regarding the control of the NCD pandemic would be divided between the responsibilities of central and regional authorities concerning the elimination of the causes of poor health, health education and reliable information about the impact of harmful addictions [26]. Obligations for eliminating the causes of poor health must include a healthy environment (including: clean air; minimising risks associated with radioactive radiation, asbestos hazards; food safety; preventing addiction to tobacco, alcohol, and other addictive substances), administering immunisations, health condition monitoring and accident prevention [27]. On the other hand, the requirement of the state’s commitment to health education concerns both the individual and the whole community equally, both in private life, at work and in public life [28]. The ECSR commits to informing the public about health risks: nicotinism, alcoholism and other harmful substances for humans, which takes place through awareness raising campaigns [26, 27].

Local and regional self-governments would enable local communities to participate in the management of the essential part of public affairs that may concern their healthy lifestyle [29]. What is even more important, local and regional authorities make decisions about their responsibilities and in the interests of their residents, including those that have direct consequences in terms of rights and health obligations. Representatives elected in democratic elections implement the principle of subsidiarity in cooperation with non-governmental organisations and institutions [30, 31]. This way, they are more effective in undertaking specific actions to combat the causes and effects of civilisation diseases.

The scope and content of obligations in the fields of promotion and protection of a healthy lifestyle that would allow a life free from NCDs is similar in most of the Council of Europe countries, which in 1985 signed the European Charter of Local Self-Government (ECLSG) [32]. To implement tasks related to combating civilisation diseases (NCDs), local and regional self-governments are entitled to establish supra-regional and cross-border cooperation. In the case of Poland, the role of local self-government bodies: commune, poviat and province, comes down to three basic tasks: prevention, protection and promotion [20]. Tasks resulting from the constitutional right to health were imposed on self-government administration bodies based on acts of international law, including the ECLSG. Therefore, they remain largely in line with the obligations imposed on government administration bodies.

The analysis of ECSR and ECHR decisions shows that the tasks entrusted to local and regional communities concerning the obligations to protect human health comes down to two spheres, positive (obligation to act) and negative (refraining from interference in the sphere of human health), that make up the health safety of the individual and protect against the effects of civilisation diseases [33, 34]. This normative structure has also functioned in the Polish legal system since Poland joined the Council of Europe in 1993. In positive terms, local and regional self-governments perform tasks on their own behalf and on their own responsibility. This consists in creating good living conditions and promoting a healthy lifestyle that allows avoiding NCDs [2, 5, 35]. Local and regional self-governments are also independent in the scope of creating pro-health living conditions for local communities, managing the local health care system, supporting preventive examinations, and are able to finance specific activities related to the promotion of physical and health activities, and pro-health attitudes (e.g. a healthy food fair) [36]. In negative terms, consisting of preventing NCDs, local self-government bodies may create acts of local law that limit the scope of rights and freedoms granted to individuals (e.g. the formation of anti-smoking zones) due to the priority of universal health protection in the social dimension [37].

### Communal governments’ tasks in the area of ​​limiting the NCDs pandemic

As in other European countries, the division of tasks in the field of the prevention of NCDs in Poland is regulated at the statutory level. A commune’s tasks in the area of protection and promotion of human health are the most fundamental ones. This is because the commune is the basic local authority in Poland that is closest to citizens and patients [38]. According to Art. 7, par. 1, item 5 of the Act on communes, satisfying the collective needs of the community in the field of health protection is one of the commune’s tasks [39]. Using the criterion of functions, these tasks can be divided into own and commissioned, while following the subject criterion, tasks in the area of individual health promotion and public health protection can be distinguished [38, 40, 41]. However, more and more often in recent years, due to the lack of funds in the central budget, the protection of children’s health against adverse effects, including improper nutrition, bad treatment, as well as sports habits [38, 42], has been transferred to communes by the Ministry of Health. Malnutrition and diseases have far-reaching effects on children’s physical health and development; they also affect their mental state because they inhibit learning progress and participation in social life, and undermine development prospects [40]. The same applies to obesity and an unhealthy lifestyle [39].

The reminder of the right to health expressed in Art. 25 of UDHR, Art. 12 of ICESCR, and in particular the right to health both in the narrow and broad sense is numerous obligations imposed on the commune by the law on social assistance [18, 33, 34]. Communes develop strategies for solving social problems, analyse social effects of NCDs, and take specific obligatory actions in the field of health protection of people who are not able to take care of their health [43]. First, communes are obliged to protect health in the social dimension, i.e. in the scope of organization of functioning of primary health care accessible to everyone, without any subjective limitations. Second, the commune acts as the payer referred to in the Act on Health Benefits [39]. Another group of laws imposes on communes obligations in the scope of ensuring sanitary safety, including ​​monitoring of the risk of NCDs at the local level. Fourth, in the scope of prevention communes are obliged to maintain cleanliness and primary health care in their areas and deal with the conditions for conducting activities in the field of environmental policy [44].

The own tasks include preventing NCDs. In the light of Art. 40, paragraph 3 of the Act on communal self-government and in the absence of regulations, the commune council may issue order regulations if it is necessary to protect the life or health of citizens and to ensure order, peace and public safety [39, 44]. In particular, they include issuance of programmes for specific areas of health protection: anti-alcohol, anti-smoking or anti-smog [45]. The commune issues such regulations based on communal strategies for solving social problems. What is more, the commune’s own tasks include activities related to the prevention and solving of alcohol-related problems, and the social integration of alcohol addicts, as well as appointing municipal commissions for solving alcohol-related problems. By way of a resolution, the commune council may establish places designated as smoke-free zones for the public to use within the commune area [44, 45].

Similarly, counteracting drug addiction is one of the commune’s own tasks [41]. It includes increasing the availability of therapeutic and rehabilitation assistance for addicts and people at risk of addiction; providing families dealing with drug addiction problems with psychosocial and legal assistance; conducting preventive, educational and training activities for solving drug problems, in particular, for children and adolescents [39, 44]. One of the most effective measures to counteract NCDs is to conduct sports and recreational activities for students, as well as activities aimed at feeding children participating in extracurricular social care and sociotherapeutic programmes. The commune’s responsibilities also include supporting the activities of institutions, non-governmental organisations and natural persons dealing with drug addiction, as well as social assistance for addicts and families of addicts affected by poverty and social exclusion, and integrating with the local community using social work and social contract [39–41].

A unique solution used in Poland after World War II that is known worldwide is an organised, public health-resort system. There are 45 health-resort communes in Poland that accept people based on medical referrals. A sanatorium is a place of relaxation and peace in which you can reduce susceptibility to NCDs. The commune’s task is to create such regulations to respect the right of all patients to privacy, peace and comfort [46]. Communes with health-resort status are a zone free of industry and tobacco smoke, and the use of mobile phones and other personal electronic devices in a sanatorium is prohibited.

In turn, in the aspect of health promotion, the commune is obliged to promote the health of pregnant women by providing detailed information on their entitlements, including health care services [40]. At a woman’s request, the commune is obliged to indicate an institution that must respect all her reproductive rights, including the right to abortion [44]. Communes, as well as other local self-government units, promote health and factors affecting it to the National Health Program [46] and the National Program for Mental Health Protection [47]. Numerous obligations imposed on the commune in the field of health promotion result from the law on environmental protection as well as the sports law, with majority of them imposing optional tasks.

### The tasks of regional authorities to limit the pandemic of NCDs

One of the tasks of regional authorities is to protect local communities against the pandemic of diseases of affluence and its consequences. In Poland, the obligation to prevent and combat diseases of affluence rests with authorities operating at district and province level [39, 44]. Unfortunately, their duties have not been distributed evenly, which means that the competences of district and province authorities overlap. District authorities may adopt a health policy which is established by the government or which follows from the District Authorities Act [48]. In this case, the representatives of such authorities act on their own initiative. They also prepare a strategy to solve health problems and fight their causes. The programme includes prospective and current actions taken by district authorities, whose aim is to promote knowledge about social, mental and spiritual health, as well as potential mechanisms for its protection [40, 49].

Regional authorities are obliged to properly shape the determinants of health and living conditions at the supra-local level [44]. They have to prevent harmful physical phenomena which affect health, such as polluted air, smog, carbon monoxide and fire, excessive noise, ionising radiation, and non-potable water [48]. Another task of district authorities is to make sure that the local community has access to basic resources which are beneficial to health. Food, shelter, clothing and a satisfying job are usually counted among these [50]. When it comes to the social aspect, district authorities have a duty to help the disabled. It includes the obligation to place sick patients in health care centres and in the case of patients with tuberculosis, follow the request of the tuberculosis clinic [48].

District authorities organise and provide services of appropriate quality in residential and nursing homes, which are adjusted to the special needs of people with mental disorders [39, 40]. The aim is to prevent such disorders and promote appropriate treatment [40, 48]. In order to protect the lives and health of residents against the consequences of domestic violence and homelessness, one of the tasks of district authorities is to provide shelter, in particular houses for pregnant women and single mothers with young children. District authorities are also obliged to provide healthcare and housing assistance for migrants, helping them to adapt and satisfying their basic health needs [41]. In certain justified cases, the district council may issue regulations, especially if it is necessary to protect the lives, health or property of citizens, to protect the environment or to maintain order, peace and public safety, given that these causes are identified on the territory of more than one commune [45]. Regional authorities have more opportunities to increase the awareness of residents, which contributes to the prevention of risks related to NCDs. In Poland, this duty rests with province authorities [40, 44]. They are obliged to carry out public tasks whose aim is to promote health at the province level, given that such tasks are not reserved to government administration authorities by relevant acts. The promotion of a healthy lifestyle and the obligation to protect human health from the disastrous consequences of NCDs would be included in a development strategy prepared by province authorities solely for this purpose [40]. Province authorities also prepare a preventive healthcare plan and alcoholism treatment programmes [49]. Interestingly, province authorities have certain instruments at their disposal which could be used to impose health security and a healthy lifestyle [47]. The extent to which authorities can interfere in the rights of residents in order to protect their health is regulated by Art. 43(2) of the Act on Province Government, according to which it is only possible “in matters of great urgency, when there is a direct threat to the public interest, in life or health-threatening situations” [44].

The basic task of regional authorities is to prevent and mitigate the consequences of NCDs, both at the individual and social level, and also to prevent stigmatisation, discrimination and a lack of respect towards sick patients [49]. The province government creates and maintains occupational health centres, whose aim is to provide preventive healthcare for workers and monitor their health [40, 44]. The tasks and competences of the province government were determined in the Social Assistance Act. Other acts order the authorities to create and manage programmes to counteract social exclusion, to create equal opportunities for the disabled, to provide social assistance and preventive healthcare, and to treat alcoholism [49]. These programmes are necessary to maintain extensive cooperation with non-governmental organisations for the promotion of a healthy diet, exercise and a healthy lifestyle [49, 51]. Depending on the needs related in particular to the number of inhabitants of a given province and the social structure of its communities, the province government creates and manages healthcare centres which provide health services in the fields of psychiatry, cardiovascular diseases and cancer [47, 49].

### Problems in the implementation of health protection and prevention of NCDs in Poland

The originators of the 1999 government, local government and healthcare reform intended to accelerate the process of decentralizing the state healthcare service, to help the local community meet their increasingly diverse health needs, to improve the availability of medical services at the local level and to enhance the quality of preventive and medical services [13, 20]. The theory was modelled on the strategies of pluralism in healthcare, the fundamentals of environmental sustainability, the principles of a free medical market and the notion of a patient’s free choice in selecting medical services offered or supervised by local communities [22, 25]. Unfortunately, in practice, the three-part administrative division into townships (gminas), counties (poviats) and voivodships at the local and regional level did not cause a significant visible improvement in healthcare [13, 38]. The diffusion of powers and the division of political and administrative competences between local governments and the central government echoes the conflict between the need to implement public health policy and the need of local communities to find effective instruments to protect their health from NCDs [13, 20, 22, 38].

Other problems that hinder the enactment of local health policies are a lack of synergy of the actions undertaken by local and regional authorities, the incompatibility of the expectations of local communities with the public policies in the healthcare sector which are the responsibility of central authorities, the mismanagement of resource allocation in the healthcare sector and the inadequate distribution of public funds allocated for combating NCDs [13, 22, 35]. Local governments make quite liberal use of the funds for NCDs that they receive from the central budget. Polish municipalities (gminas) receive special-purpose subsidies from the state budget only for selected health care tasks. Funds for non-communicable diseases (NCDs) awarded as subsidies from the state budget are rather low, although they are being systematically increased. While in 2017, total expenditure on health in all municipalities in Poland was 142,953,000 PLN (32,929,000 EUR), in 2018 it increased by 2.8% to a total of 146,928,000 PLN (33,844,000 EUR) [51]. In 2018, 96.5% of the subsidies for ongoing tasks commissioned to municipalities were allocated to projects carried out by assistance centres, care services and specialised care services, and the payment of health insurance fees for people receiving social assistance benefits. Some municipalities combine health care with spending on education, including children’s health education (for example, promoting sport and preventing obesity among school-aged children) [51, 52]. It is worth noting, however, that local initiatives in the form of the citizens’ participatory budget, that is the part of the budget that can be decided by residents, are directly related to NCDs only to a minimal degree. This means that the tasks connected with the prevention of NCDs compete with other tasks of the municipal authorities [20, 42].

When it comes to counties (poviats), they receive the largest funding of all local levels of government. However, these funds are systematically reduced due to the political conflict that arose in 2015 between the central government and the local authorities [40, 52]. For this reason, county authorities have to resort to using additional funds from local taxes year after year. In 2017, the central budget subsidy for NCD-related objectives was 610,749,000 PLN (140,686,000 EUR), while in 2018 it was reduced by 16.3% to 510,954,000 PLN (117,698,000 EUR) [51]. Expenses for the prevention of NCDs accounted for as much as 18.5% of the counties’ financial resources. As much as 2.3% of funds obtained by the counties were allocated to general hospitals, sobering-up stations and health policy programs. The largest part of county expenditure in 2018 (19.2%) was attributable to investment projects in emergency medical services [51].

In voivodships, financial expenditure in 2018 on health care and on combating NCDs was 759,024,000 PLN (174,841,000 EUR), with as much as 68.6% being incurred by financial expenditure in general hospitals, 10.4% by psychiatric treatment, 9.1% by general activities and 5.1% by emergency medical services [51, 52]. Expenses for health care make up 12.8% of all voivodship expenses and have increased by 184,109 00 PLN (42,409,000 EUR) compared to 2017, including 17% for the prevention of NCDs. Voivodships obtain funds from the central budget; in 2018 they received special-purpose subsidies of 37,723,000 PLN (868,950 EUR) only for investments associated with NCDs in general hospitals and for emergency medical services and psychiatric treatment [51, 52]. A significant part of the special-purpose subsidy (36.2%) of the voivodship is also absorbed by expenses related to paying health insurance fees for the poorest and health benefits for individuals who are not under the obligation to pay health insurance premiums [51].

In addition to the issue of redistributing public funds, another problem is the insufficient participation of the local community in the management of healthcare entities at the local level [20–22]. What is unique in Poland is the existence, apart from the local and regional governments, of professional councils, including the councils of doctors, dentists, rescuers, nurses and midwives. Furthermore, a high degree of institutionalization in the area of public healthcare causes patients to be reluctant to voice their ideas and postulates on preventing and combating NCDs. Representatives of the medical professions in post-communist countries, as a rule, are not interested in allowing representatives from outside their group to make decisions that fall within their competence [13, 48]. Many years of ignoring the voice of local communities have resulted in a lack of interest among residents in the proper management of local hospitals and other healthcare facilities, as well as low expectations in terms of the quality of NCD programs [51]. In practice, the influence of local communities on the staffing of institutions dealing with NCDs is minor, and the only representatives of local communities in such facilities are patient ombudsmen. This claim is also confirmed by the absence of references to healthcare policy in the electoral platforms of the vast majority of local political parties.

Unfortunately, apart from obligatory actions, only one in four Polish local governments financed its own initiatives for the health of its residents [51, 52]. There are many factors that contribute to this. Firstly, since the 1999 reform, the reimbursement of medicines, the number of treatments in hospitals, the salaries of doctors and the amount of funds spent on promoting a healthy lifestyle have been decided by the government in Warsaw, not by local and regional governments. Secondly, the relationships between local and regional authorities are another problem. Due to the dispersion of competences between local and regional communities, joint supra-regional and interregional initiatives are very rare [39]. The exceptions are cross-border initiatives financed from international funds and carried out in cooperation with local governments from Germany, the Czech Republic, Lithuania and Slovakia, which seek to improve the health of the population living in the border region [13, 51, 52]. Thirdly, representatives of local and regional authorities often do not want to cooperate with each other, because they usually come from different, mutually hostile political parties, and their statutory tasks have been created as competitive rather than complementary.

Representatives of local and regional authorities are not easily persuaded by non-epidemiological potential health threats. According to the 2016 Supreme Audit Office report, access to local programmes is limited, and local government units, often despite having identified the needs, do not have the funds [20, 39, 40]. Even in the richest municipalities and counties, health expenditure does not exceed 3% of the total annual local government funds, while it is estimated that the optimal level of expenditure should exceed 6%. In the budget of the capital city of Warsaw, only 2% of the entire budget was channelled into health [20, 42, 51]. Most of the municipal funds allocated to NCDs are intended for compulsory programs to combat drugs and alcohol abuse, while other healthcare needs are not adequately met: the promotion of knowledge about emergency medical services, co-financing campaigns and dental assistance [40–42]. All of these actions should be implemented under public health policies, which the WHO defines as ‘decisions, plans, and actions that are undertaken to achieve specific healthcare goals within a society’ [8, 13]. Therefore, 30 years after the political transformation, due to the lack of local public health policies, Polish citizens have ceased to believe in having real influence on their local decision-makers and are trying to protect their health with their own resources. This is a peculiar variation of the British concept of new localism, which in Poland is the equivalent of the local community confining themselves in a narrow and limited local space [22, 25]. It is rather an expression of distrust of the concept of health policy, not of one outside the local territory, but a distrust that is a reaction to the lack of effective, local health policy.

## Conclusion

Although complete elimination of NCDs is impossible without coordinated efforts from the entire international community, it is necessary that both local and regional authorities at least control the causes of NCDs and monitor their subsequent development [2, 8, 47]. For this purpose, the following conclusions were drawn. First of all, representatives of local and regional communities would in their activities take into account the so-called right to the health paradox, which indicates that as the wealth of the society increases, the health care needs of members of local and regional communities do not drop, but grow [6]. Protection of citizens of a given state against the negative impact of NCDs requires intensified legislative, preventive and promotional actions launched not only in terms of intergovernmental cooperation, but also at a local and regional level and through inter-region cooperation [2, 19, 40]. This pushes for synergies and intensification of activities taken up by central governments, local and regional authorities in accordance with the principle of subsidiarity set forth in the ECLSG [31].

What is the most significant, limiting the proliferation of NCDs also requires, in vertical terms, sound financial efforts on the part of local authorities [42]. The representatives of these communities are responsible for encouraging the inhabitants of their communities to change their lifestyle: change their eating habits, increase physical activity, remain outdoors more frequently, reduce stimulants, and apply prevention, namely undergo regular medical check-ups and provide the body with all the necessary nutrients [2, 52]. In addition, health campaigns as well as health and prophylactic programmes absorb significant amounts from the budgets of local governments and thus require the acquisition of sponsors, cooperation with medical, pharmaceutical and non-governmental organisations as well as substantive support from the WHO and other international organisations [13].

In practice, what is even more important, the excessive division of competences and the dispersion of resources between regional and local authorities may, however, hamper the effective treatment and prevention of NCDs. Taking Poland as an example, in the conditions of diffusion of the decision-making process at the local and regional level, we can observe a lack of holistic thinking about the health of local communities [25]. The multitude of entities authorised to implement healthcare policy is not favourable for successfully combating NCDs. A lack of cooperation between the central government, which carries out an independent policy on public health, and local authorities, which use only a portion of the public funds allocated to health, obstructs the debate on priorities at the societal level and does not encourage residents to take a stand on how to allocate extra funds [22, 23]. It is essential to eliminate the discrepancies in health policies between politicians and administration at all three levels — central, regional and local — which the concept of new localism makes possible [21, 22]. Also, without developing proper health habits among the members of the local communities and persuading them that their dedicated commitment to local affairs will not be wasted, a wide-ranging fight against NCDs at the local level will not be feasible.

## Data Availability

All data is contained within the manuscript file.

## Abbreviations

ECHR: European Court of Human Rights
ECLSG: European Charter of Local Self-Government
ECSR: European Committee of Social Rights
EU: European Union
ICCPR: International Covenant on Civil and Political Rights
ICESCR: International Covenant on Economic, Social and Cultural Rights
NCDs: Non communicable diseases
UDHR: Universal Declaration of Human Rights
WHO: World Health Organization
